# Real-world lymphoma cohort in an HIV-endemic setting: cohort design, epidemiological features and impact of implementing novel classification standards

**DOI:** 10.1186/s12885-026-15807-8

**Published:** 2026-04-02

**Authors:** Lillian F. Andera, Dharshnee R. Chetty, Zainab Mohamed, Diana Oelofse, Jenna Bailey, Karryn Brown, Girisha Panchoo, Katherine Antel, Kudakwashe Simba, Gerdien Kritzinger, Sumaiya Cassim, Amsha Ramburan, Jessica J. Opie, Vernon J. Louw, Estelle Verburgh

**Affiliations:** 1 Division of Clinical Haematology, Department of Medicine, Faculty of Health Sciences, University of Cape Town and Groote Schuur Hospital, Cape Town, South Africa; 2 Division of Anatomical Pathology, Department of Pathology, Faculty of Health Sciences, University of Cape Town and National Health Laboratory Services, Groote Schuur Hospital, Cape Town, South Africa; 3 Division of Radiation Oncology, Department of Radiation Medicine, Faculty of Health Sciences, University of Cape Town and Groote Schuur Hospital, Cape Town, South Africa; 4 Department of Histopathology, Manchester University NHS Foundation Trust, Manchester, UK; 5 Division of Haematology, Department of Pathology, Faculty of Health Sciences, University of Cape Town and National Health Laboratory Services, Groote Schuur Hospital, Cape Town, South Africa; 6 Hollings Cancer Institute, Medical University of South Carolina, Charleston, USA

**Keywords:** Reclassification, Registry, Lymphoma, HIV, South Africa

## Abstract

**Background:**

Lymphoma real-world observational data and accurate diagnostic systems are lacking in low-resource HIV-endemic settings. We established a new lymphoma registry to generate internationally comparable, clinically validated data, with up-to-date disease classification. We also describe our findings on aggressive B-cell lymphoma.

**Methods:**

The descriptive retrospective cohort included patients ≥ 13 years of age newly diagnosed with lymphoma from 2005 to 2020. Patients were enrolled at a single site in a registry with hierarchical groupings to capture, interrogate, and subtype lymphoma diagnoses. These were standardised on the most recent version of the World Health Organisation Classification of Haematolymphoid Tumours (WHO-HAEM5) and correlated with the novel International Consensus Classification of mature lymphoid neoplasms (ICC). Differences due to nomenclature and diagnostic category were annotated.

**Results:**

The cohort consisted of 2354 incident lymphoma cases; 1891 (80.3%) non-Hodgkin lymphoma and 463 (19.7%) Hodgkin lymphoma (HL). Twenty-one lymphoma *NOS* cases were excluded due to inadequate specimen for standardised subclassification. Overall reclassification according to WHO-HAEM5 was 25.8% (*n* = 608). Notable nomenclature differences between WHO-HAEM5 and ICC included 44 (1.9%) transformations of indolent B-cell lymphomas; also 857 (36.4%) lymphoid proliferations and lymphomas associated with immune deficiency/dysregulation due to HIV (33.1%) and EBV (31.8%). EBV-association was highest among HL cases, of which 77 (50.3%) were HIV reactive.

**Conclusion:**

We report here the impact of adopting international lymphoma classification standards in an HIV-endemic setting. Our findings highlight the persistent prevalence of large B-cell lymphoma, raise concern around inadequate HIV suppression as a potential ongoing driver of disease in our setting, and provide further evidence for EBV-associated HL as a distinct subtype.

## Background

Real-world observational data have been identified as important adjuncts to bridge the knowledge gap in cancer studies and are under-utilised in low-resource settings [1–3]. The process of generating internationally comparable, clinically validated data in these settings is challenging. The accurate classification of lymphoma is crucial for effective treatment and prognostication, yet the diagnostic pathway in the sub-Saharan African (SSA) context is complicated by resource limited pathology services, and the more pressing clinical demands of the human immunodeficiency virus (HIV) and tuberculosis endemic environment [4–9]. Most of the historical lymphoma diagnostic pathology and epidemiology studies were conducted by working groups from high income countries (HIC) [10–15]. Comparatively few of these studies were independently generated from within the SSA region [16–22].

Population-based cancer registries in low- and middle-income countries (LMIC), where they exist, contribute data classified within broad topographical categories to the collaborative platforms of the World Health Organisation (WHO): International Agency for Research on Cancer (IARC) and International Association of Cancer Registries (IACR) [4, 9, 13, 23]. A few specialised lymphoma cohorts have, nonetheless, been established in recent decades; intermittently reporting tentatively disaggregated lymphoma data to the international scientific community. In the SSA region these cohorts were mostly representative of single institutions and focused on the impact of HIV as the primary driver of lymphoma in the early anti-retroviral treatment (ART) era [16, 17, 19, 20]. These studies were, furthermore, mostly pathology-based, with data classified along the directives of earlier versions of the WHO Classification of Haematolymphoid Tumours, hereafter referred to as WHO-HAEM, and in some instances utilised obsolete morphology-based classification [11, 12, 15].

The continually updated WHO-HAEM multi-modal framework is the standard for accurate contemporary diagnosis of lymphoma, concurrent to a second classification standard, the International Consensus Classification of mature lymphoid neoplasms (ICC) [24–27]. Both classification systems, WHO-HAEM5 and ICC, share fundamental concepts of disease classification that integrate clinical, pathological and molecular data [27, 28]. Careful insertion of lymphoma cases into the hierarchical framework of the modern diagnostic standard, establishes the platform for subsequent refinement and enrichment of downstream diagnostic categorisation, and even in a low resource setting, enables globally comparable data collection by cancer registries [29, 30].

With these new insights, the University of Cape Town Lymphoma Working Group (UCT LWG), a multidisciplinary team of pathologists, haematologists, database developers and data capturers affiliated with Groote Schuur Hospital in the Western Cape province of South Africa, set out to develop a new specialised lymphoma registry empowered to deliver high quality clinically validated data categorised according to up-to-date standards. We report here on cohort design, epidemiological features pertinent to an HIV-endemic, resource-restricted setting, and on the operational impact of implementing novel classification systems, with a focus on aggressive lymphoma. The UCT LWG lymphoma cohort encompasses all lymphoma cases diagnosed from 2005 in this institution and continues to operate prospectively from the Division of Clinical Haematology.

## Methods

### Study design

**Fig. 1:**
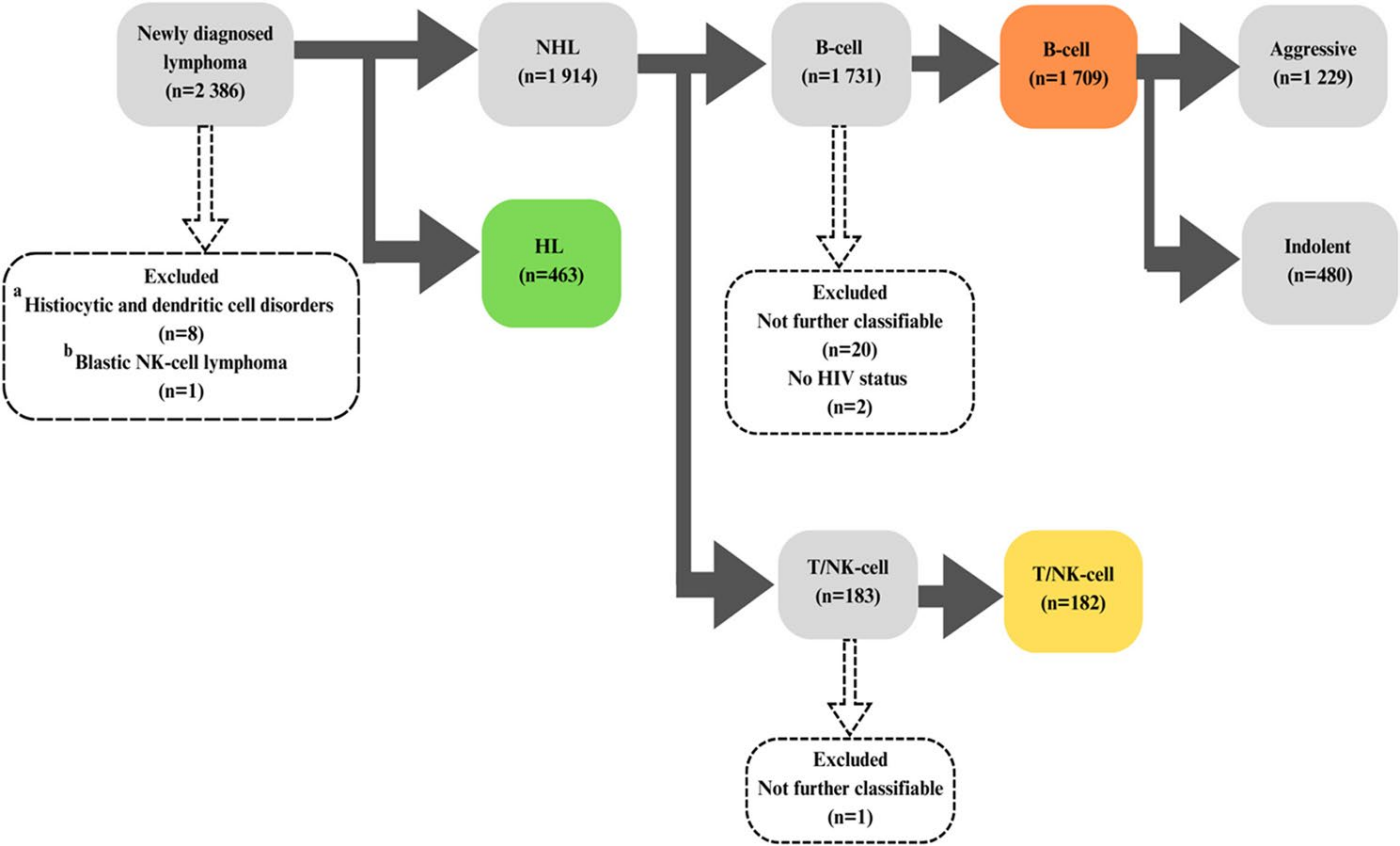
The exclusive cohort of newly diagnosed lymphoma cases according to the WHO-HAEM5 and clinical presentation. ^a, b^ cases reclassified from lymphoid to myeloid

The Groote Schuur Hospital Haematology Patient Registry, a large parent cohort of haematological malignancies, was established in 2018 by the UCT Division of Clinical Haematology. This observational cohort is systematically enriched with retrospective data covering all the major categories of haematological neoplasms and is prospectively maintained. The registry utilises a secure web-based Research Electronic Data Capture platform (REDCap software 2024, version 13.7.9 Vanderbilt University, Nashville, TN, USA) developed and hosted at UCT to collect and store patient information [31, 32]. For this first retrospective descriptive study of the lymphoma sub-cohort, the UCT LWG identified all lymphoma patients ≥ 13 years of age with a new lymphoma diagnosis managed at Groote Schuur Hospital between 1 st January 2005 to 31 st December 2020. The inclusion of the adolescent group, 13 years and older, is reflective of local hospital admission practice [33]. Data were abstracted from clinical records stored at Groote Schuur Hospital’s Division of Clinical Haematology and the Division of Radiation Oncology. Patients were matched with their laboratory records originating from the ISO accredited Divisions of Anatomical Pathology and Haematology of the National Health Laboratory Services. All patients with a new lymphoma diagnosis in the study period were included once. Duplicate entries were avoided by including patients with an earlier or concurrent indolent lymphoma diagnosis under the respective nomenclature befitting transformations from indolent B-cell lymphomas. Likewise in cases with more than one type of high-grade lymphoma in the study period, only the first diagnosis was included while in patients with discordant lymphomas, only the aggressive lymphoma was included. Other exclusions leading to the cohort of interest are highlighted in Fig. 1.

### Study population

The catchment area comprises both urban and rural settings in the Western Cape province of South Africa. Groote Schuur Hospital is a 975-bed regional academic treatment centre that receives around half of all tertiary, adult public sector referrals in the province (South Africans and resident foreign nationals) [34, 35]. Patients with medical insurance are usually treated in the private sector and seldom diagnosed at Groote Schuur Hospital. According to the latest South African general household survey conducted in 2021, approximately 47.9% of the Western Cape population of 7.2 million is dependent on public health facilities [36].

### Data collection

Demographic and baseline clinical data were obtained from paper and electronic records. Patient characteristics such as sex and age were obtained from the patient clinical management and booking system of the South African National Department of Health, Clinicom (Citrix 2024 Cloud Software Group, Inc. South Africa). The lymphoma diagnosis, date of diagnosis, Epstein-Barr virus (EBV) and HIV status were obtained from the National Health Laboratory Services information systems, DISA (DISA*LAB 2017 Johannesburg, South Africa) (archived) and TrakCare (TrakCare Lab 2012, version L6.10, InterSystems Corporation, Morningside, South Africa) (active), the unified healthcare information systems, and electronic medical records tool for healthcare professionals in South Africa’s public healthcare facilities.

### Disease categorisation

#### Hierarchical classification

The REDCap data dictionary categorisation pathway of the lymphoma cohort was modelled on a hierarchical taxonomy of lymphoid neoplasms [37]. This universally accepted methodology, embedded in the WHO-HAEM4R and International Classification of Diseases for Oncology 3rd edition (ICD-O-3), systematically categorises diseases from general to increasingly specific entities and subtypes aligned with their respective International Classification of Diseases 10th revision (ICD-10) [26, 38, 39].

#### Creating a baseline standardised cohort

Patients in our retrospective cohort were historically diagnosed and classified according to their contemporaneous WHO iteration set out in either the WHO-HAEM3, WHO-HAEM4, or WHO-HAEM4R [24–26]. To that end, the primary researcher first standardised, and where required, reclassified all cases to a baseline WHO-HAEM4R category [26]. Classifications were based on a primary source morphology diagnosis obtained from soft tissue, bone marrow, peripheral blood or body cavity fluid. To reach the most accurate downstream diagnosis, morphology reports and all available ancillary modalities [immunohistochemistry (IHC) stains, immunophenotype on flow cytometry, karyotype, fluorescence in-situ hybridisation (FISH) and gene rearrangement studies], were interrogated, integrated and validated with the respective clinical case notes. Pathology reports that contained incomplete or differential diagnoses were subjected to review and escalated to the UCT LWG histopathologists and haematopathologists (DRC, DO, GP, SC, JJO, EV) to complete subclassification.

Cases were next delineated into non-Hodgkin lymphoma (NHL) and Hodgkin lymphoma (HL); and NHL into B-cell or T/NK-cell lineage neoplasms. Clinico-pathological features before treatment and proliferation rate according to a Ki-67 expression ≥ 40% triggered classification work-up for aggressive B-cell lymphoma and therefore also guided the assignment of cases to an indolent or aggressive clinical group in our registry [40–43]. Whilst some lymphoma cases were concluded upstream in more general categories, most were fully subclassified downstream to specific subtypes.

#### Cohort enrichment with retrospective investigations in high-grade lymphoma

Prior to WHO-HAEM4 our pathology department followed a proto-version of an “essential for diagnosis approach” with limited IHC and molecular diagnostics to refine subtyping of aggressive/high-grade B-cell lymphomas (HGBL) and blastoid morphologies. Following the implementation of WHO-HAEM4 in 2008, the introduction of cell-of-origin classification, Hans algorithm and the concept of double-hit HGBL, supplementary investigations were introduced incrementally to routine operational laboratory methodology [25, 44–46].

Ki-67 expression by IHC > 40–70% (reported as a percentage of positive tumour nuclei) in conjunction with supportive morphology and immunophenotype is widely recognised to be compatible with many aggressive lymphomas such as the majority of diffuse large B-cell lymphoma (DLBCL) and guided workup in our cohort. Ki-67 > 70–80% strongly supported a highly proliferative/poor prognosis subgroup of aggressive lymphomas. Ki-67 **≥** 90–100% is characteristic of Burkitt lymphoma or Burkitt-type biology and was integrated with morphology features, immunophenotype and cytogenetics/MYC testing to conclude diagnosis [40–42].

To achieve diagnostic refinement across the study cohort, the research team performed additional retrospective IHC stains (CD10, BCL6, MUM1, BCL2, C-MYC) and FISH investigations (*MYC*,* BCL2*,* BCL6*) on cases that lacked adequate aggressive lymphoma work-up [39]. Affiliated sub-cohort studies provided some of the additional laboratory data (IHC stains, cytogenetic and FISH results, and EBV-related investigations). These studies included (i) DLBCL, not otherwise specified (*NOS*) which examined cell-of-origin subtypes, EBV co-infection and CD4 counts in the setting of high HIV prevalence; (ii) EBV viral load in newly diagnosed HL; and (iii) molecular testing and CD4 counts in patients with newly diagnosed HIV-Burkitt lymphoma [47–49].

#### Impact of cohort standardisation and reclassification, specifically HGBL, to WHO-HAEM5 and ICC

To assess the operational impact of implementing novel classification standards we reclassified our WHO-HAEM4R standardised cohort according to updated guidelines of WHO-HAEM5 and ICC and computed the number of specialised investigations required to facilitate reclassification. WHO-HAEM5 was utilised as the denominator informing temporal changes. This study did not assess the clinical or financial impact of revised diagnoses.

#### Temporal analysis

To assess temporal changes in our own laboratory approach to refined diagnosis of aggressive lymphoma specifically, we audited our diagnostic algorithm used in the diagnosis of HGBL and enumerated the number of FISH investigations required to achieve refined downstream subtyping. We disaggregated and correlated data across the three previous WHO-HAEM versions.

### Data analysis and statistical methods

StataCorp. 2023 *Stata Statistical Software: Release 18* (StataCorp LLC, College Station, TX, USA) was used for analysis. Categorical variables were described by frequencies and percentages. Age at diagnosis was described by medians and interquartile ranges as data were non-parametric. Age and sex were compared in the HIV-negative and HIV-positive groups by the Mann–Whitney U test and chi-square test, respectively. Focus areas included numbers and percentages of lymphoma cases by subtype, EBV status, HIV status, and cytogenetics performed over 16 consecutive years. The infographics were created using the online design tool Canva (https://www.canva.com/). Time trends were displayed using bar graphs created in Microsoft Excel.

## Results

### Establishing the lymphoma cohort

Cohort design and workflow are illustrated in Fig. 1. Overall, 2386 lymphoma cases consecutively diagnosed between 1 st January 2005 and 31 st December 2020 were analysed. Isolated cases that lacked HIV status were excluded. Among other early exclusions were 21 lymphoma - *NOS* cases and instances where patients with insufficient data for definitive subclassification were lost to follow-up. Table 1 shows the final cohort reclassified to WHO-HAEM5. Additional disaggregation for HIV status represents the WHO-HAEM5 category lymphoid proliferation and lymphomas associated with immune deficiency/dysregulation (IDD), in the setting of HIV. For 19 patients known to have had more than one type of high-grade lymphoma during the study period, only the first diagnosis was analysed. There were 44 patients who presented with transformations of indolent B-cell lymphoma (whether synchronous or with a prior diagnosis of indolent lymphoma); these were only tallied in their aggressive/large cell lymphoma WHO-HAEM5 category. The distribution of cases according to WHO-HAEM4R is provided in supplementary Table 1.

**Table 1:** Baseline characteristics of lymphoma patients diagnosed between 2005–2020 at Groote Schuur Hospital, categorised according to the WHO-HAEM5 and highlighting lymphoma in the setting of HIV infection

	Total No.	HIV-	HIV+	*p*-value
Lymphoma Cohort	2354	1575	779	
Males	1222 (51.9%)	822 (52.2%)	400 (51.4%)	0.700
Females	1132 (48.1%)	753 (47.8%)	379 (48.7%)	
Age (years), median (IQR)	47.6 (35.0–62.0)	56.6 (41.0–67.3)	38.3 (32.5–45.3)	< 0.001
**Non-Hodgkin lymphoma**	**1891 (80.3%)**	**1265 (80.3%)**	**626 (80.4%)**	**0.981**
**Tumour-like lesions with B-cell predominance**				
IgG4-related disease	1 (0.04%)	1 (0.06%)	-	
KSHV/HHV8-associated multicentric Castleman disease	76 (3.2%)	6 (0.4%)	70 (9.0%)	
**Mature B-cell neoplasms**				
**Pre-neoplastic and neoplastic small lymphocytic proliferations**				
Monoclonal B-cell lymphocytosis	2 (0.08%)	2 (0.1%)	-	
Chronic lymphocytic leukaemia	179 (7.6%)	176 (11.2%)	3 (0.4%)	
Small lymphocytic lymphoma	22 (0.9%)	21 (1.3%)	1 (0.1%)	
**Splenic B-cell lymphomas and leukaemias**				
Hairy cell leukaemia	18 (0.8%)	18 (1.1%)	-	
Splenic marginal zone lymphoma	15 (0.6%)	15 (1.0%)	-	
Splenic B-cell lymphoma/leukaemia with prominent nucleoli	3 (0.1%)	3 (0.2%)	-	
**Lymphoplasmacytic lymphoma**				
Lymphoplasmacytic lymphoma	20 (0.8%)	20 (1.3%)	-	
**Marginal zone lymphoma**				
Extra nodal marginal zone lymphoma of MALT	55 (2.3%)	52 (3.3%)	3(0.4%)	
Nodal marginal zone lymphoma	11 (0.5%)	11 (0.7%)	-	
**Follicular lymphoma**				
Follicular lymphoma	154 (6.5%)	153 (9.7%)	1 (0.1%)	
**Cutaneous follicle centre lymphoma**				
Primary cutaneous follicle centre lymphoma	1 (0.04%)	1 (0.06%)	-	
**Mantle cell lymphoma**				
Mantle cell lymphoma	47 (2.0%)	45 (2.9%)	2 (0.3%)	
**Transformations of indolent B-cell lymphomas**				
Transformations of indolent B-cell lymphomas	44 (1.9%)	41 (2.6%)	3 (0.4%)	
**Large B-cell lymphomas**				
Diffuse large B-cell lymphoma, NOS	588 (25.0%)	415 (26.3%)	173 (22.2%)	
DLBCL Germinal centre B-cell subtype	270	189	81	
DLBCL Activated B-cell subtype	197	145	52	
DLBCL, NOS unspecified^a^	121	81	40	
T-cell/histiocyte-rich large B-cell lymphoma	17 (0.7%)	15 (1.0%)	2 (0.3%)	
EBV-positive DLBCL	49 (2.1%)	16 (1.0%)	33 (4.2%)	
Plasmablastic lymphoma	95 (4.0%)	7 (0.4%)	88 (11.3%)	
Primary large B-cell lymphoma of immune-privileged sites	24 (1.0%)	17 (1.1%)	7 (0.9%)	
CNS	15	9	6	
Testis	9	8	1	
Intravascular large B-cell lymphoma	1 (0.04%)	1 (0.06%)	-	
Primary mediastinal large B-cell lymphoma	24 (1.0%)	24 (1.5%)	-	
Mediastinal grey zone lymphoma	3 (0.1%)	3 (0.2%)	-	
High-grade B-cell lymphoma, NOS^b^	50 (2.1%)	11 (0.7%)	39 (5.0%)	
**Burkitt lymphoma**				
Burkitt lymphoma	194 (8.2%)	23 (1.5%)	171 (22.0%)	
**KSHV/HHV8-associated B-cell lymphoid proliferations and lymphomas**				
Primary effusion lymphoma	5 (0.2%)	1 (0.06%)	4 (0.5%)	
KSHV/HHV8-positive DLBCL	1 (0.04%)	-	1 (0.1%)	
**Lymphoid proliferations and lymphomas associated with IDD (non-HIV)**			-	
Burkitt lymphoma, post-transplant setting	2 (0.08%)	2 (0.1%)	-	
DLBCL Germinal Centre B-cell subtype, post-transplant setting	2 (0.08%)	2 (0.1%)	-	
DLBCL Activated B-cell subtype, post-transplant setting	6 (0.3%)	6 (0.4%)	-	
Peripheral T-cell lymphoma, post-transplant setting	1 (0.04%)	1 (0.06%)	-	
**Mature T-cell and NK-cell neoplasms**				
**Mature T-cell and NK-cell leukaemias**				
T-cell prolymphocytic leukaemia	5 (0.2%)	5 (0.3%)	-	
T-large granular lymphocytic leukaemia	2 (0.08%)	1 (0.06%)	1 (0.1%)	
NK-large granular lymphocytic leukaemia	1 (0.04%)	1 (0.06%)	-	
Adult T-cell lymphoma/leukaemia (HTLV-1-associated)	2 (0.08%)	1 (0.06%)	1 (0.1%)	
Sezary Syndrome	1 (0.04%)	1 (0.06%)	-	
**Primary cutaneous T-cell lymphomas**				
Mycosis fungoides	48 (2.0%)	47 (3.0%)	1 (0.1%)	
Primary cutaneous γδ T-cell lymphoma	1 (0.04%)	1 (0.06%)	-	
Primary cutaneous anaplastic large cell lymphoma	4 (0.2%)	3 (0.2%)	1 (0.1%)	
Primary cutaneous CD4 + small/medium T-cell LPD	1 (0.04%)	1 (0.06%)	-	
Primary cutaneous peripheral T-cell lymphoma, NOS	4 (0.2%)	4 (0.3%)	-	
**Intestinal T-cell and NK-cell lymphoid proliferations and lymphomas**				
Enteropathy-associated T-cell lymphoma	1 (0.04%)	1 (0.06%)	-	
**Hepatosplenic T-cell lymphoma**				
Hepatosplenic T-cell lymphoma	5 (0.2%)	5 (0.3%)	-	
**Anaplastic large cell lymphoma**				
ALK-positive Anaplastic large cell lymphoma	14 (0.6%)	11 (0.7%)	3 (0.4%)	
ALK-negative Anaplastic large cell lymphoma	23 (1.0%)	17 (1.1%)	6 (0.8%)	
Anaplastic large cell lymphoma, unspecified^c^	2 (0.08%)	-	2 (0.3%)	
**Nodal T-follicular helper (TFH) cell lymphoma**				
Nodal TFH cell lymphoma, angioimmunoblastic-type	13 (0.6%)	12 (0.8%)	1 (0.1%)	
**Other Peripheral T-cell lymphomas**				
Peripheral T-cell lymphoma, NOS	41 (1.7%)	35 (2.2%)	6 (0.8%)	
Other peripheral T-cell lymphoma, unspecified^d^	4 (0.2%)	4 (0.3%)	-	
**EBV-positive NK-cell and T-cell lymphomas**				
EBV positive nodal T- and NK-cell lymphoma	1 (0.04%)	1 (0.06%)	-	
Extra nodal NK/T-cell lymphoma	8 (0.3%)	5 (0.3%)	3 (0.4%)	
**Hodgkin lymphoma**	**463 (19.7%)**	**310 (19.7%)**	**153 (19.6%)**	**0.981**
Classic Hodgkin lymphoma	436 (18.5%)	284 (18.0%)	152 (19.5%)	
Nodular sclerosis Hodgkin lymphoma	228	181	47	
Mixed cellularity Hodgkin lymphoma	63	35	28	
Lymphocyte depleted Hodgkin lymphoma	11	7	4	
Lymphocyte-rich Hodgkin lymphoma	15	10	5	
Hodgkin lymphoma, unspecified^e^	119	51	68	
Nodular lymphocyte predominant Hodgkin lymphoma	27 (1.1%)	26 (1.7%)	1 (0.1%)	

Bold text represents hierarchical classification of lymphoma, showing major disease categories and their respective families

*CNS* central nervous system, *HIV* human immune deficiency virus, *EBV* Epstein-Barr virus, *HHV-8* human herpesvirus 8, *HTLV-1* Human T-lymphotropic virus 1, *IDD* Immune deficiency/dysregulation, *KSHV* Kaposi sarcoma herpesvirus, *LPD* Lymphoproliferative disorder, *MALT* Mucosa-associated lymphoid tissue, *NOS* Not otherwise specified

^a^Based on inconclusive immunohistochemistry; this category includes DLBCL Germinal centre B-cell and DLBCL Activated B-cell subtype

^b^Includes two high-grade B-cell lymphoma, NOS and 43 suspected NOS but not enough staining done or had insufficient FISH performed to confirm a true NOS; other five cases were based on morphology and some immunohistochemistry but could not be classified as DLBCL or Burkitt lymphoma

^c^Includes ALK-positive and ALK-negative anaplastic large cell lymphoma. ALK immunostaining was not done in this category

^d^Not enough stains done to further subclassify

^e^Diagnoses made on bone marrow only (*n* = 45), tissue histology only (*n* = 16), both bone marrow and tissue histology (*n* = 58). These could not be subclassified further due to inconclusive immunohistochemistry

### Baseline epidemiological characteristics

The final cohort comprised 2354 newly diagnosed patients − 1891 (80.3%) NHL and 463 (19.7%) HL (Table 1). The proportional distribution for indolent B-cell lymphoma was 22.5% and aggressive B-cell lymphoma 57.8%. Indolent B-cell lymphoma was a rare diagnosis (1.03%) in the setting of HIV, compared to a high proportion of aggressive B-cell lymphoma cases (76.1%). The median age for all patients was 47.6 years and significantly lower in people living with HIV (PLWH) − 38.3 years (IQR 32.5-45.3) compared to the HIV negative group 56.6 years (IQR 41.0–67.3); *p* < 0.001. Median age of indolent B-cell lymphoma patients was 63.9 years (IQR 55.0–71.3), compared to 46.1 years (IQR 35.5–60.2) for aggressive B-cell varieties *p* < 0.001.

### Reclassifications

Table 2 illustrates the impact of cohort standardisation to WHO-HAEM4R and the overall number of cases that required further review and/or additional diagnostic modalities to be reclassified to WHO-HAEM5–25.8% (*n* = 608). The evolution in disease categorisation, as informed by sequential changes from WHO-HAEM4R to WHO-HAEM5 and ICC, is captured in supplementary Table 2. We highlight the reclassification of 44 (1.9%) cases as transformations of indolent B-cell lymphomas and 862 (36.6%) cases assigned to the IDD group with three-part integrated nomenclature.

**Table 2:** Impact of cohort standardisation, with number of cases requiring further review and/or additional diagnostic modalities to reclassify from WHO-HAEM4R to WHO-HAEM5 (n=608)

Cases that required retrospective revision for standardisation to WHO-HAEM4R, and reclassification to WHO-HAEM5	No.	WHO-HAEM5 type and/or subtype destination of reclassified cases	No.2
Burkitt-like lymphoma	4	Burkitt lymphoma	11
Intermediate between Burkitt lymphoma & DLBCL	2		
HGBL	3		
Burkitt lymphoma vs intermediate between Burkitt lymphoma & DLBCL	2		
Burkitt-like lymphoma	3	Burkitt leukemia, variant	26
Burkitt lymphoma	19		
HGBL	1		
HGBL possibly Burkitt lymphoma	1		
Burkitt lymphoma vs DLBCL (immunoblastic)	1		
HGBL with features intermediate between Burkitt lymphoma & DLBCL	1		
DLBCL	134	DLBCL, ABC	142
Highly suggestive LBCL	1		
LBCL	2		
HGBL	2		
DLBCL, *NOS*	3		
DLBCL	150	DLBCL, GCB	180
DLBCL, *NOS*	5		
DLBCL with plasmablastic differentiation	2		
Suggestive of LBCL	2		
Suggestive of HGBL	1		
Atypical B-cell lymphoproliferation	1		
Blastoid mantle cell lymphoma	2		
DLBCL vs Follicular lymphoma grade 3	3		
HGBL	11		
HGBL favours Burkitt lymphoma	1		
Intermediate between Burkitt lymphoma & DLBCL	2		
B-cell non-Hodgkin lymphoma	2	DLBCL, *NOS*	135
B-cell lymphoma	3		
DLBCL vs Nodular sclerosing classic Hodgkin lymphoma	1		
DLBCL	106		
HGBL	6		
LBCL	10		
NHL	1		
Suggestive of lymphoma	1		
Suspected transformation THRLBCL to DLBCL	3		
DLBCL vs blastoid mantle cell lymphoma	1		
DLBCL with plasmablastic differentiation	1		
DLBCL	11	EBV positive DLBCL	20
DLBCL, *NOS*	5		
HGBL	2		
EBV positive HGBL vs lymphomatoid granulomatosis	1		
EBV positive HGBL	1		
Burkitt-like lymphoma	10	HGBL, *NOS*	27
Burkitt lymphoma	4		
HGBL vs Burkitt lymphoma	3		
Intermediate between Burkitt lymphoma & DLBCL	9		
DLBCL, GCB	1		
DLBCL, *NOS*	9	Primary LBCL of immune privileged sites, Testis	9
B-cell lymphoma	1	Primary LBCL of immune privileged sites, CNS	2
DLBCL, GCB	1		
Plasmacytoma	1	Plasmablastic lymphoma	1
DLBCL or Primary mediastinal large B-cell lymphoma	1	Primary mediastinal large B-cell lymphoma	2
HGBL	1		
Hodgkin lymphoma vs Anaplastic large cell lymphoma	1	Classic Hodgkin lymphoma, *unspecified*	1
Interfollicular lymphoma	1	Mixed cellularity classic Hodgkin lymphoma	1
Histiocytic sarcoma vs T-cell lymphoma	1	PTCL, *NOS*	3
Cutaneous T-cell lymphoma	1		
T-cell lymphoma	1		
PTCL, *NOS*	4	Primary cutaneous PTCL, *NOS*	4
Large B-cell entities preceded by, or with synchronous transformation, from a small B-cell entity (n=44)		Transformations of indolent B-cell lymphomas (n=44)	
DLBCL with histological background suggestive of FL	21	DLBCL transformed from FL	21
DLBCL with histological background suggestive of MZL	2	DLBCL transformed from MZL	2
DLBCL with histological background suggestive of LPL	1	DLBCL transformed from LPL	1
DLBCL with histological background suggestive of MALT	3	DLBCL transformed from MALT	3
DLBCL with histological background suggestive of CLL	1	DLBCL transformed from CLL	1
High-grade B-cell lymphoma with synchronous FL	5	HGBL (suspected transformation from synchronous FL)	5
High-grade lymphoma with preceding low-grade lymphoma	11	HGBL transformed from low-grade lymphoma	11
Total number	608	Total number	608

*ABC* activated B-cell, *CNS* central nervous system, *DLBCL* diffuse large B-cell lymphoma, *GCB* germinal center, *CLL* chronic lymphocytic leukemia, *EBV* Epstein-Barr virus, *FL* follicular lymphoma, *HGBL* high grade B-cell lymphoma, *LBCL* large B-cell lymphoma, *LPL* lymphoplasmacytic lymphoma, *MALT* Mucosa-associated lymphoid tissue, *MZL* Marginal zone lymphoma, *NOS *not otherwise specified, *PTCL* Peripheral T-cell lymphoma, THRLBCL T-cell/histiocyte-rich large B-cell lymphoma

### Implementation of cytogenetic tests

**Fig. 2:**
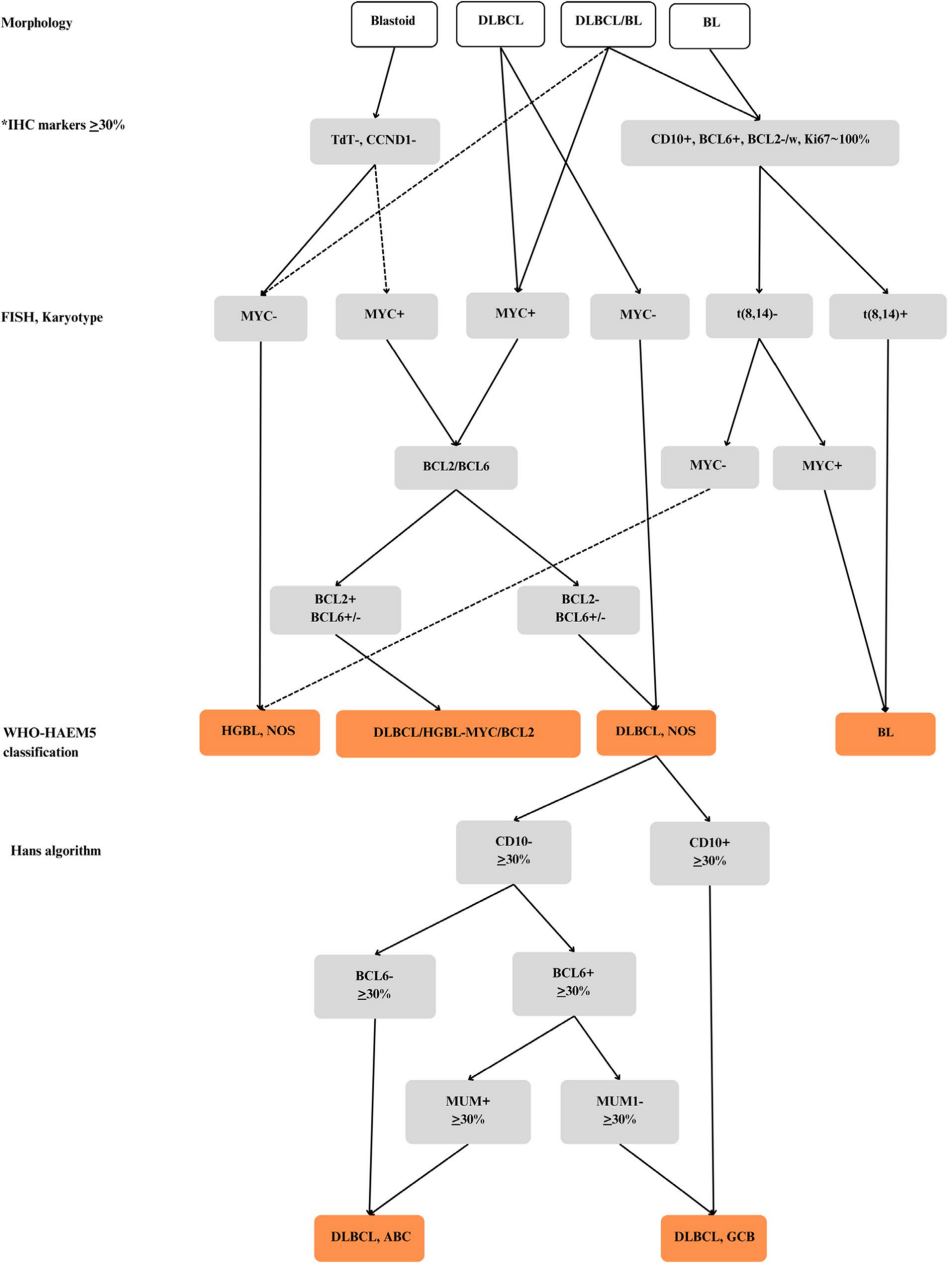
Standardised approach to differential diagnosis of aggressive B-cell lymphomas on tissue or lymph nodes at Groote Schuur Hospital. *The diagnostic modalities for BL or DLBCL with leukaemic involvement include immunophenotype characterisation by flow cytometry. BL Burkitt lymphoma, DLBCL diffuse large B-cell lymphoma, HGBL high-grade B-cell lymphoma, *NOS* not otherwise specified

**Fig. 3:**
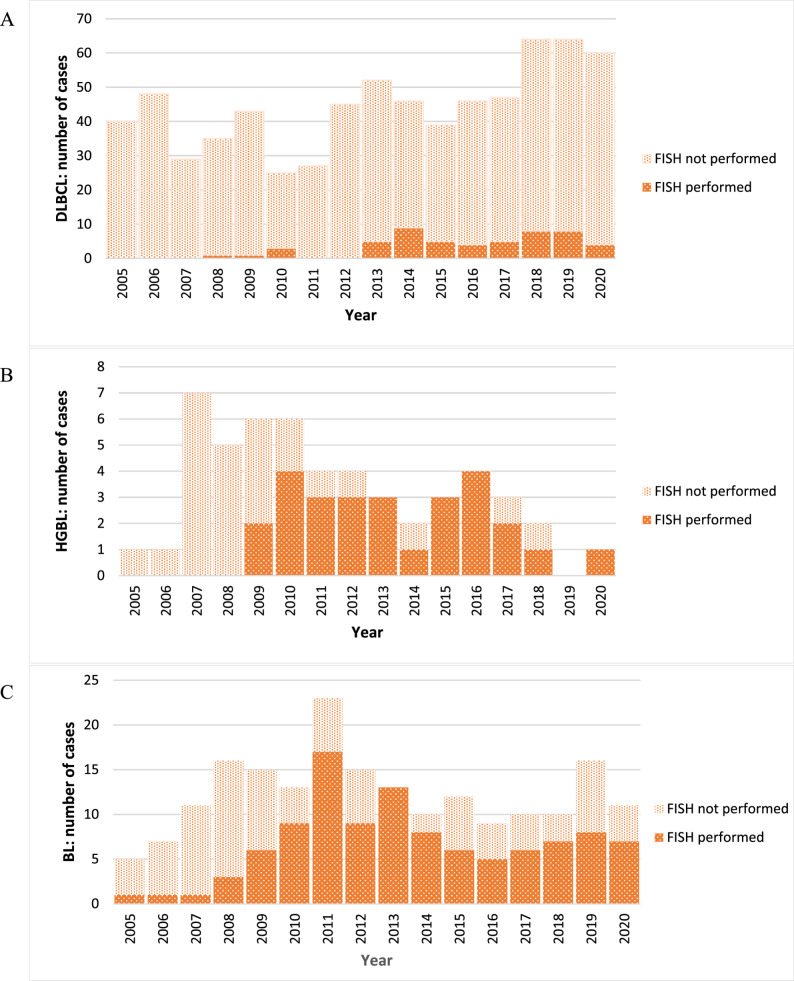
Temporal changes in utilisation of FISH for disease categorisation and standardisation to WHO-HAEM4R and WHO-HAEM5 (2005–2020): aggressive/high proliferative lymphomas. **A** DLBCL, diffuse large B-cell lymphoma (*n* = 710), **B** HGBL, *NOS* high-grade B-cell lymphoma, not otherwise specified (*n* = 52) and **C** BL, Burkitt lymphoma (*n* = 196)

Figure 2 illustrates the standardised approach to differential diagnosis of aggressive B-cell lymphomas at Groote Schuur Hospital. Figure 3 represents the overall impact of temporal changes in utilisation of FISH in disease categorisation and standardisation to WHO-HAEM4R and WHO-HAEM5 (2005–2020) for aggressive/high proliferative lymphomas. Additional FISH investigations carried out to refine HGBL diagnoses gradually increased over time and were performed for 53 (7.5%) DLBCL, 26 (50.0%) HGBL, *NOS* and 107 (54.6%) Burkitt lymphoma cases. Although 186 (15.3%) of all HGBL had at least one FISH investigation, only 16 cases with a confirmed positive IHC MYC (> 40%) result, had all three FISH probes *MYC*-*BCL2*-*BCL6*, performed. An assessment of temporal trends showed that the FISH testing kick-started by the prior sub-cohort studies, fostered and validated the utility of more comprehensive testing on a routine basis [47, 48]. The WHO-HAEM4R entity DLBCL *NOS* with *MYC*-*BCL6* gene rearrangements was found in a single case, but after WHO-HAEM5 reclassification, no ‘double hit lymphomas’ with *MYC*-*BCL2* gene rearrangements were identified.

### EBV cohort

EBV stain results from in-situ hybridisation with EBV encoded RNA (EBER-ish) and latent membrane protein 1 (LMP1) are summarised in Table 3 [47, 49, 50]. In total 770 (32.7%) cases of the cohort were tested. EBV positivity was found in 31.8% (*n* = 245), with a significant proportion concurrently HIV reactive (*n* = 167, 68.2%; *p* < 0.001). The majority of EBV-positive DLBCL cases (67.3%) were among PLWH. Among plasmablastic lymphoma cases (92.6% in PLWH), 82.6% (*n* = 46) were EBV positive. Although only a small sample, 13.2% (*n* = 10), of KSHV/HHV8-associated multicentric Castleman disease cases (92.1% in PLWH) were analysed for EBV, 60% were EBV-associated and therefore triple positive for EBV, HHV8 and HIV. EBV results were likewise only available for a small sample of Burkitt lymphoma 25.3% (*n* = 49), with comparatively few EBV positive (22.4%); the majority in PLWH (88.1%). Newly diagnosed HL in PLWH (*n* = 153) was also very commonly EBV-associated (*n* = 77, 50.3%). We additionally, identified a rare example of EBV-associated nodular lymphocyte predominant HL.

**Table 3:** Lymphoid proliferation and lymphomas associated with immune deficiency/dysregulation in the setting of HIV, diagnosed between 2005-2020 at Groote Schuur Hospital Cape Town: cases tested for EBV

	**EBV Tested**	**EBV+**	**HIV-**	**HIV+**	*p*-value
EBV Lymphoma Cohort	**770**	**245**	**78**	**167**	**<0.001**
Non-Hodgkin lymphoma	**524 (68** **.** **1%)**	**129 (52** **.** **7%)**	**39 (50** **.** **0%)**	**90 (53** **.** **9%)**	**0** **.** **570**
Tumour-like lesions with B-cell predominance					
KSHV/HHV8-associated multicentric Castleman disease	10 (1.3%)	6 (2.4%)	-	6 (3.6%)	
Large B-cell lymphomas					
Diffuse large B-cell lymphoma, NOS	279 (36.2%)	-	-	-	
DLBCL Germinal centre B-cell subtype	139	-	-	-	
DLBCL Activated B-cell subtype	130	-	-	-	
DLBCL, NOS unspecified^1^	10	-	-	-	
T-cell/histiocyte-rich large B-cell lymphoma	6 (0.8%)	-	-	-	
EBV-positive DLBCL	49 (6.4%)	49 (20.0%)	16 (20.5%)	33 (19.8%)	
Plasmablastic lymphoma	46 (6.0%)	38 (15.5%)	3 (3.8%)	35 (21.0%)	
Primary large B-cell lymphoma of immune-privileged sites					
CNS	4 (0.5%)	2 (0.8%)	-	2 (1.2%)	
Testis	4 (0.5%)	-	-		
Intravascular large B-cell lymphoma	1 (0.1%)	-	-	-	
Primary mediastinal large B-cell lymphoma	7 (0.9%)	-	-	-	
Mediastinal grey zone lymphoma	3 (0.4%)	-	-	-	
High-grade B-cell lymphoma, NOS^2^	10 (1.3%)	-	-	-	
Burkitt lymphoma					
Burkitt lymphoma	49 (6.4%)	11 (4.5%)	2 (2.6%)	9 (5.4%)	
KSHV/HHV8-associated B-cell lymphoid proliferations/lymphomas					
Primary effusion lymphoma	3 (0.4%)	3 (1.2%)	1 (1.3%)	2 (1.2%)	
KSHV/HHV8-positive DLBCL	1 (0.1%)	-	-	-	
Lymphoid proliferations and lymphomas associated with IDD (non-HIV)					
Burkitt lymphoma, post-transplant setting	1 (0.1%)	-	-	-	
DLBCL Germinal Centre B-cell subtype, post-transplant setting	2 (0.3%)	-	-	-	
DLBCL Activated B-cell subtype, post-transplant setting	6 (0.8%)	6 (2.4%)	6 (7.7%)	-	
Anaplastic large cell lymphoma					
ALK-positive Anaplastic large cell lymphoma	5 (0.6%)	-	-	-	
ALK-negative Anaplastic large cell lymphoma	10 (1.3%)	-	-	-	
Nodal T-follicular helper (TFH) cell lymphoma					
Nodal TFH cell lymphoma, angioimmunoblastic-type	6 (0.8%)	5 (2.0%)	4 (5.1%)	1 (0.6%)	
Other Peripheral T-cell lymphomas					
Peripheral T-cell lymphoma, NOS	14 (1.8%)	2 (0.8%)	2 (2.6%)	-	
Other peripheral T-cell lymphoma, unspecified^3^	2 (0.3%)	1 (0.4%)	1 (1.3%)	-	
EBV-positive NK-cell and T-cell lymphomas					
EBV positive nodal T- and NK-cell lymphoma	1 (0.1%)	1 (0.4%)	1 (1.3%)	-	
Extra nodal NK/T-cell lymphoma	5 (0.6%)	5 (2.0%)	3 (3.8%)	2 (1.2%)	
Hodgkin lymphoma	**246 (31** **.** **9%)**	**116 (47** **.3%)**	**39 (50** **.** **0%)**	**77 (46** **.** **1%)**	**0** **.** **570**
Classic Hodgkin lymphoma	231 (30.0%)	115 (46.9%)	38 (38.5%)	77 (46.1%)	
Nodular sclerosis Hodgkin lymphoma	128	52	19	33	
Mixed cellularity Hodgkin lymphoma	36	26	8	18	
Lymphocyte depleted Hodgkin lymphoma	7	5	1	4	
Lymphocyte-rich Hodgkin lymphoma	9	4	2	2	
Hodgkin lymphoma, unspecified^4^	51	28	8	20	
Nodular lymphocyte predominant Hodgkin lymphoma	15 (1.9%)	1 (0.4%)	1 (1.3%)	-	

*CNS* central nervous system, *EBV* Epstein-Barr virus, *HHV8* human herpesvirus-8, *IDD* immune deficiency/ dysregulation, *KSHV* Kaposi sarcoma herpes virus,* NOS* not otherwise specified

^1^Based on inconclusive immunohistochemistry; this category includes DLBCL Germinal centre B-cell subtype and DLBCL Activated B-cell subtype

^2,3,4^Not enough stains done on tissue histology to further subclassify or diagnoses made on bone marrow

### HIV cohort

HIV prevalence was 33.1% and ART status at lymphoma diagnosis included: ART naïve [*n* = 334, (42.9%)]; on ART and virally suppressed [*n* = 285, (36.6%)]; and on ART, but virally unsuppressed [*n* = 160, (20.5%)]. The most frequent HIV-associated lymphoma entities were DLBCL, *NOS* [*n* = 173 (22.2%)]; Burkitt lymphoma [*n* = 171 (22.0%)]; and classic HL [*n* = 152 (19.5%)]. Primary large B-cell lymphomas of immune privileged sites were rare. Most low-grade NHL B-cell entities were HIV non-reactive; splenic B-cell lymphomas/leukaemia and lymphoplasmacytic lymphoma were notably absent, whereas rare cases of chronic lymphocytic leukaemia/small lymphocytic lymphoma, marginal zone lymphoma, follicular lymphoma and mantle cell lymphoma were HIV reactive. Subtype analysis of HL showed that nodular sclerosis HL cases were the most common among PLWH. Nodular lymphocyte predominant HL was a rare entity (1 of *n* = 27). Finally, of the 182 T-cell and NK-cell leukaemia and lymphomas (mature, cutaneous and other), a small proportion [*n* = 25, (3.2%)] presented in PLWH.

## Discussion

In this study from an HIV-endemic developing country, we demonstrate how rigorous lymphoma classification could be established in a real-world diagnostic registry by gradual implementation of WHO-HAEM and ICC directives. The impact of reclassifying to ICC was lower than WHO-HAEM5, with fewer disparities compared to the conversion from WHO-HAEM4R to the WHO-HAEM5. Data disaggregation revealed that the most frequent groups reclassified were the high-grade varieties of lymphoma, notably HGBL (> 50% of cases) and cases now assigned to an IDD group with three-part integrated nomenclature as endorsed by international working groups concerned with immunodeficiency-associated lymphoproliferative disorders [27, 51]. This latter category, that comprises all post-transplant lymphoproliferative disorders, HIV-positive and EBV tumour positive cases, contrasts with the ICC that presently retains the previously traditional approach. Ultimately, apart from HGBL, we found reasonable comparability between WHO-HAEM4R, WHO-HAEM5 and ICC. This agrees with data published by a large prospective HIC cohort reporting only 0.8% major diagnostic differences [52]. We, furthermore, identified only a single case of HGBL with *MYC*-*BCL6* gene rearrangements that, in a departure from previous WHO-HAEM4R methodology, would be categorised as DLBCL, *NOS* according to WHO-HAEM5, and as a defined stand-alone subtype by ICC. Similar to another South African cohort, HGBL with *MYC*-*BCL2* gene rearrangements was not identified in our cohort [53].

Another difference between WHO-HAEM5 and ICC is the recognition of transformation of indolent B-cell lymphoma as a distinct disease group [27, 28]. In our cohort, we were mostly comfortably able to identify cases of indolent B-cell lymphomas that transformed to high-grade. However, some instances of *de novo* high-grade lymphoma, with background features *morphologically resembling transformation from indolent B-cell lymphoma*, generated a degree of diagnostic uncertainty, especially where the lack of sophisticated molecular diagnostics was prohibitive for deeper characterisation. Of note also, is the annotation of residual/unmasked discordant indolent B-cell lymphomas (with bone marrow involvement) following treatment of high-grade lymphomas.

Earlier regional studies that mostly focussed on lymphoma prevalence, highlighted the prominence of high-grade lymphoma and formerly rare varieties, with HIV as the primary driver [11, 16, 17, 19, 20]. The most prevalent lymphoma entities among PLWH in our cohort agree with data reported from HIC and confirm the ongoing prominence of large B-cell lymphoma [54]. On interrogation of the clinical features of HL, we found that it frequently behaved in a “clinically aggressive” fashion not dissimilar to other established histology driven clinically aggressive high-grade entities [55]. HIV prevalence for this study cohort was slightly lower than the range (37% to 66%) reported in earlier cohorts in South Africa [16, 17, 20]. The significantly younger age at presentation for PLWH with lymphoma [median 38.3 years (IQR 32.5–45.3)] is in stark contrast to the HIV-negative patients in our cohort [median 56.6 years (IQR 41.0–67.3)] and the difference is even more striking when compared to the median age at presentation of lymphoma cohorts in HIC; for example, the United Kingdom [median 69.9 years (IQR 59.1–78.3)], USA [median 62 years (range, 18–99)], Australia and New Zealand [median 64.3 years (IQR 52.1–73.5)] [52, 56, 57]. More than half of the subpopulation of PLWH were ART naïve, or on ART but virally unsuppressed at lymphoma diagnosis highlighting viral non-suppression as an important potential driver of lymphomagenesis. It is important to note that in South Africa, HIV plasma viral loads are not routinely measured in ART naïve patients as a cost saving measure in the public health system.

The analysis of EBV testing data in our lymphoma cohort indicated a high overall EBV positivity and illuminated significant concurrent HIV reactivity. The WHO-HAEM5 entity EBV-positive DLBCL, typically a disease of older patients in HIC, occurs in comparatively younger patients in our setting, and frequently occurs in PLWH. Important to note is that the highest EBV prevalence was among HL cases with concurrent HIV reactivity, highlighting a distinct subgroup in our cohort. Therefore, the tentative EBV characterisation in our cohort supports theorisation around the increasing prominence of virally driven cancers in LMIC [27, 58, 59]. Although EBV testing was not performed in all our lymphoma cases, which may have introduced selection bias, on balance our results argue for the potential utility of routinely incorporating EBV testing in lymphoma diagnostic algorithms, in select settings [27, 28, 59, 60].

Similarly, the karyotypes for some Burkitt lymphoma cases were found to be frequently and unusually complex in the case of HIV-associated Burkitt lymphoma [48]. The availability of karyotypes in these instances obsoleted the need for *MYC* testing by FISH. This stands in contrast to the WHO-HAEM5 diagnostic algorithm for HGBL that advocates for further testing despite a *MYC* negative result. Nonetheless, the distinction between the HGBL entities DLBCL and Burkitt lymphoma, both frequently HIV-associated and clinically aggressive, but with different treatment modalities, remain of clinical importance in our setting. Our results underscore the importance of FISH analysis as a crucial adjunct to diagnostic clarity in high-grade varieties of B-cell lymphoma and reveal rare instances of double and triple viral oncogene or viral vector association. In LMIC, bespoke evidence-based diagnostic algorithms for HGBL are ultimately needed to direct the selective use of costly complimentary techniques, including FISH studies, and should be reserved for cases where results are imperative to guide and optimise therapy [61–65]. This explains why FISH testing in our cohort is remarkably low starting at WHO-HAEM4 implementation. Many of these cases were retrospectively investigated by another sub-study in this lymphoma cohort using WHO-HAEM4R [47].

The solid hierarchical framework of the WHO-HAEM5 and ICC enabled incorporation of high-quality real-world data maximising available information and minimising impact of incomplete data [66]. Challenges in our setting include scarce technical expertise, limited access to and the financial cost of advanced instrumentation and bioinformatics support. The relatively high reclassification rate illustrates how in-house operational challenges in a resource restricted setting, were ameliorated by incremental introduction of novel diagnostics in support of progressive sophistication in lymphoma diagnosis and classification. In HIC, representing around a fifth of the world’s population and benefiting from wider implementation of universal healthcare access, the categorisation of some lymphoma entities along the more comprehensive ICC diagnostic pathways, may be more achievable [28]. Both WHO-HAEM5 and ICC, nonetheless, provide improved classification methodology and can be implemented to a reasonable level of downstream refinement, provided relatively basic laboratory modalities are available.

Limitations of this study, include instances of sample insufficiency for FISH testing among some HGBL cases and insufficient IHC stains performed to achieve subclassification. Contingent on clinically extreme presentations, which are not rare in the SSA setting, bias may also be introduced by the omission of comprehensive diagnoses based on isolated pre-terminal samples. Additionally, missing clinical data and early deaths that preclude further diagnostic investigations led to exclusion of some cases. The high reclassification rate in this study may at least be partly attributed to additions and developments within the sequential versions of the WHO-HAEM [24–27]. NHL cases frequently required additional IHC stains or complementary tests to reach refined diagnoses or to upgrade to disease entities previously defined as provisional entities [1, 25]. Despite these limitations, this is the first study that comprehensively describes the population of lymphoma patients in our institution with disaggregated subtyping. Strengths of this study include the large sample size, single site integrated diagnosis and categorisation standardised to the WHO-HAEM5 providing real-world data that allows comparability to other centres, local and international [27].

## Conclusions

This study takes the first steps to highlight the complexities and challenges encountered to implement refined diagnosis and classification of lymphoma in an HIV and tuberculosis endemic LMIC setting. It also advocates for improvement in cost-effective diagnostic and classification algorithms appropriate for LMIC settings [6, 67]. Additionally, it supports the development of regional specialised cancer registries and provides a platform that fosters much needed translational research. Finally, from a public health standpoint, we hope that it may yield actionable guidance for equitable healthcare strategy development in our local context.

## Data Availability

The datasets generated and/or analysed during the current study are available from the corresponding author on reasonable request.

## Abbreviations

ART: Antiretroviral therapy
EBV: Epstein-Barr virus
FISH: Fluorescence in-situ hybridisation
HGBL: High-grade B-cell lymphoma
HIC: High income countries
HIV: Human immune deficiency virus
HL: Hodgkin lymphoma
ICC: International Consensus Classification of mature lymphoid tissues
IDD: Lymphoid proliferations and lymphomas associated with immune deficiency/dysregulation
IHC: Immunohistochemistry
LMIC: Low- and middle-income countries
NHL: Non-Hodgkin lymphoma
PLWH: People living with HIV
SSA: Sub-Saharan Africa
UCT: University of Cape Town
WHO-HAEM: World Health Organisation Classification of Haematolymphoid Tumours
